# Supplementary material for: Post-COVID impairment of memory T cell responses to community-acquired pathogens can be rectified by activating cellular metabolism
Source: bioRxiv. 2026 Jan 2:2025.12.31.697156. Preprint. [Version 1] doi: 10.64898/2025.12.31.697156 (PMC12776401; doi:10.64898/2025.12.31.697156)
Supplement: 1 [file NIHPP2025.12.31.697156V1-supplement-1.pdf]

Supplementary Figures

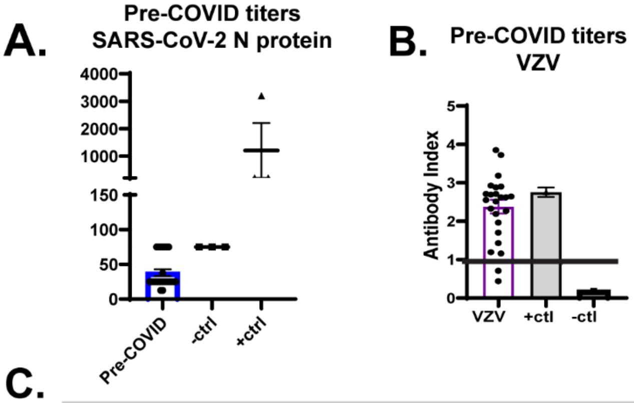

C.

Pathogen	Protein Ag	Format	Location, fn (in pathogen)
Influenza A virus, H1N1 (IAV)	Neuraminidase (NA)	13-17mers w/ 11aa overlap	Surface protein, viral release
Staphylococcus aureus (SA)	Hemolysin A (hla)	15-16mers w/10aa overlap	Secreted protein, RBC lysis
Varicella-zoster virus (VZV)	ORF4	20mers w/10aa overlap	Virion tegument,viral replication

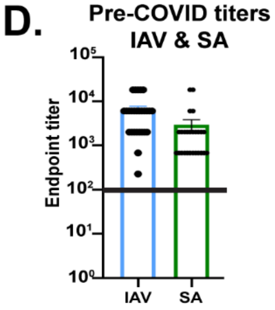

Figure S1: Pre-COVID antibody titers, VZV/SA/IAV antibody titers, antigen descriptions.

A.) All subjects were negative for exposure to COVID-19 by anti-SARS-CoV-2 N protein (Nucleocapsid) serology. -ctrl: pre-2019 samples. +ctrl: patients diagnosed with long COVID after positive PCR test. B.) 93% of study subjects were positive for VZV antibody titers upon enrollment. All were confirmed to have been vaccinated or exposed to natural infection in childhood. -ctrl and +ctrl were provided with the manufacturer’s kit (ACRO Biosystems). C.) Description of antigens used in stimulation from VZV, IAV, and SA. D.) All subjects were positive for IAV and SA IgG by serology. Horizontal lines in B & D represent limit of detection.

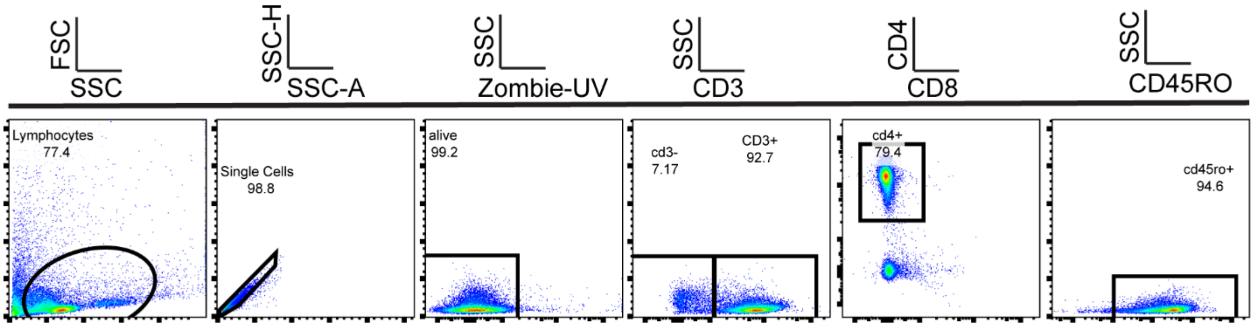

Figure S2: CD4 memory T cell purity after magnetic bead negative selection.

PBMCs were stimulated with VZV-Orf4 peptides and CD4+CD45RO+ cells were purified by negative selection using a Miltenyi CD4 memory T cell purification kit. Samples were approximately 92% CD3+ T cells, of which 80% were CD4+.

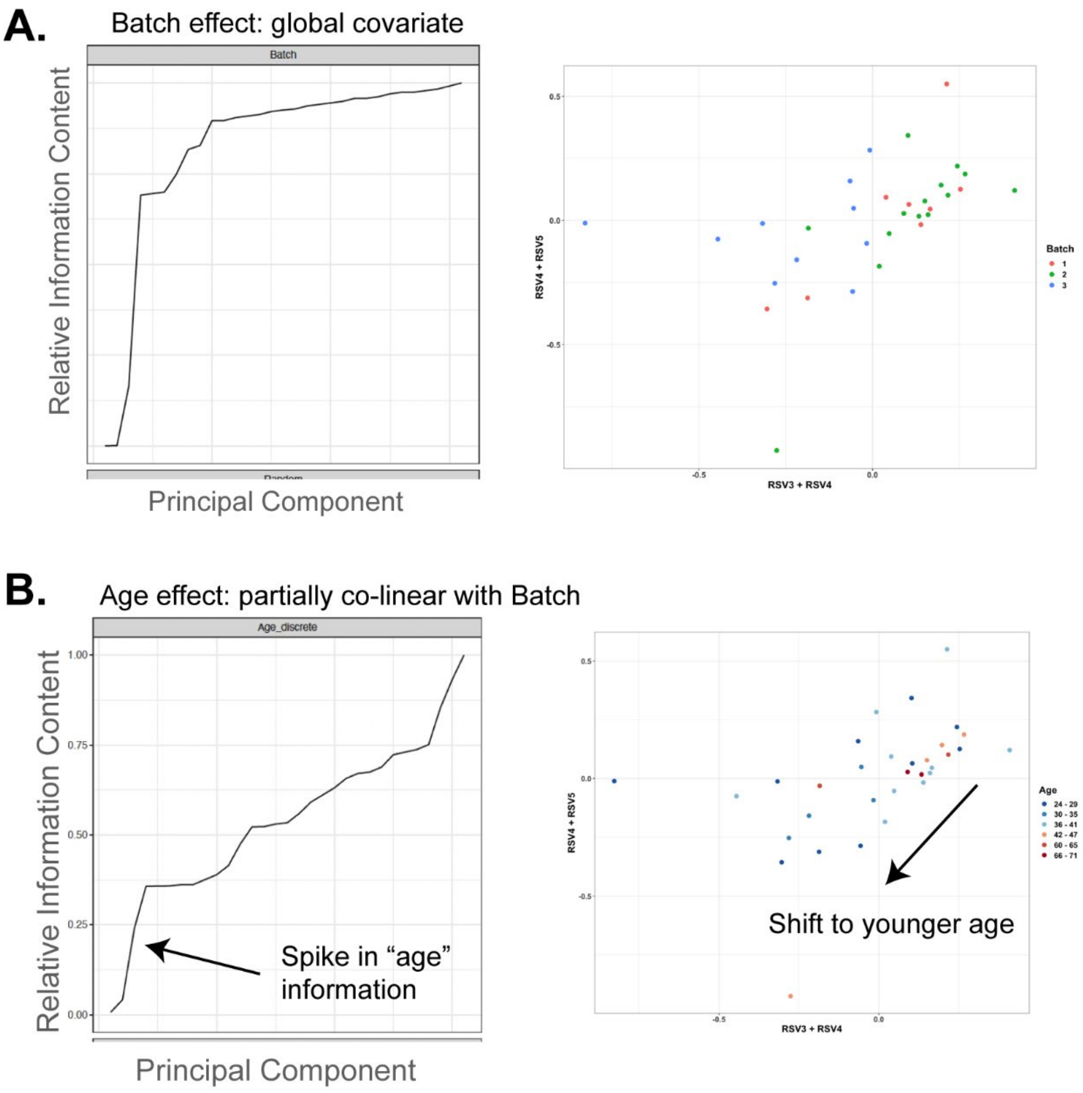

Figure S3: PCA analysis of pre- and post-COVID RNA-Seq data uncover batch and age effects as main global covariates.

A.) Traditional PCA analysis identified batch effects as principal components describing statistical variation. B.) Age effects are similarly identified among the top PCs and are partially co-linear with batch effects.

A.

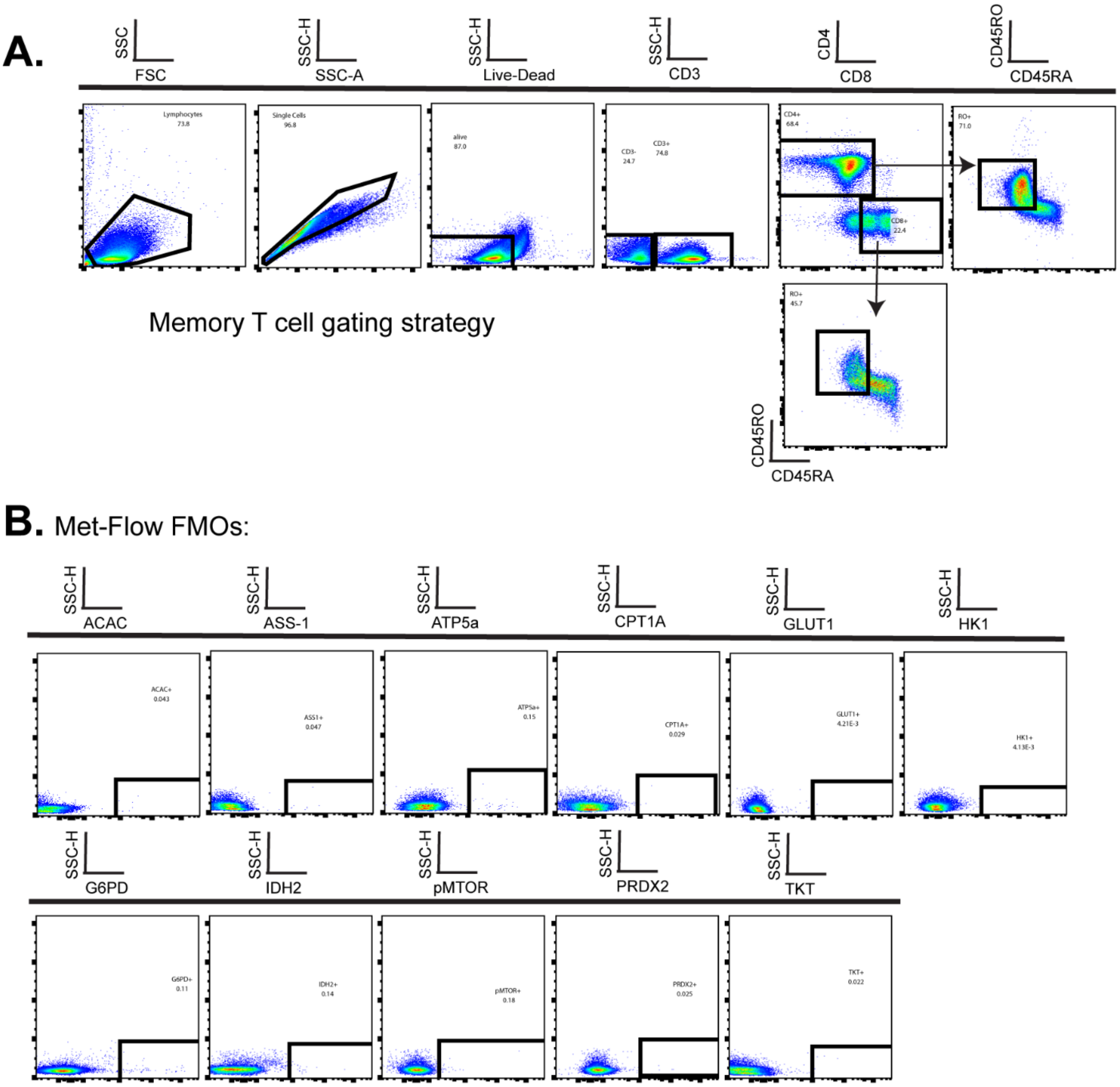

B. Met-Flow FMOs:

Figure S4: Flow cytometry gating strategies.

A.) Strategy for gating CD4 and CD8 memory T cells. B.) Fluorescence minus one (FMO) gating for Met-Flow enzymes.

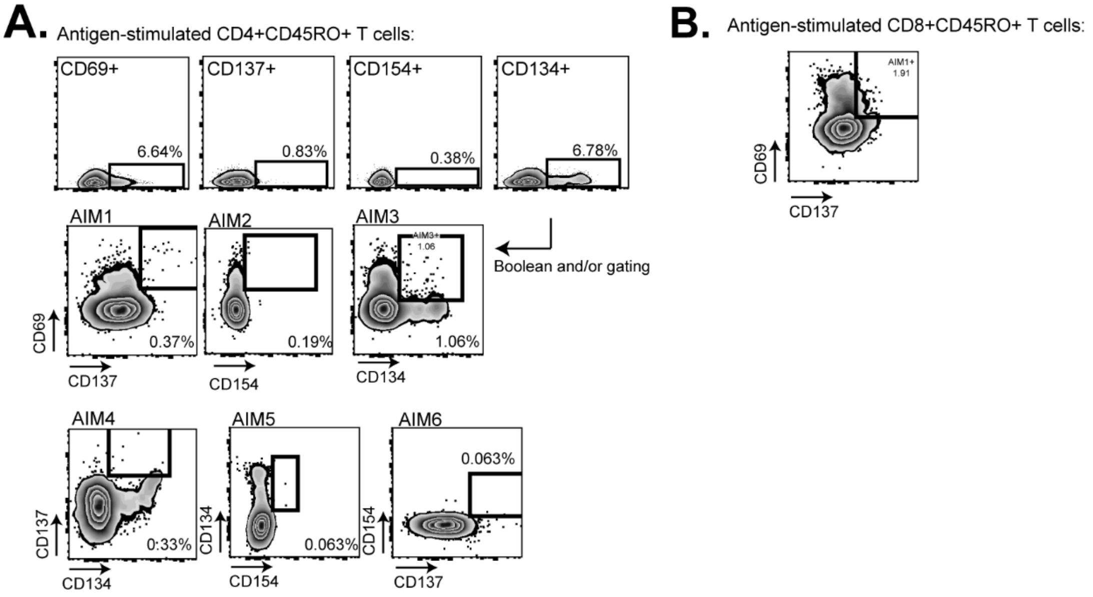

Figure S5: Gating strategy for AIM+ CD4 and CD8 memory T cells.

A.) Boolean gating strategy was applied for identifying AIM+ CD4+CD45RO+ T cells. Cells were gated on CD69/CD137/CD154/CD134+ as in the top row before applying Boolean and/or gating (combination gates) in Flowjo. The 6 AIM combinations shown were concatenated together and metabolic enzyme expression analyzed within the concatenated population. B.) Gating strategy for AIM+CD8 memory T cells.

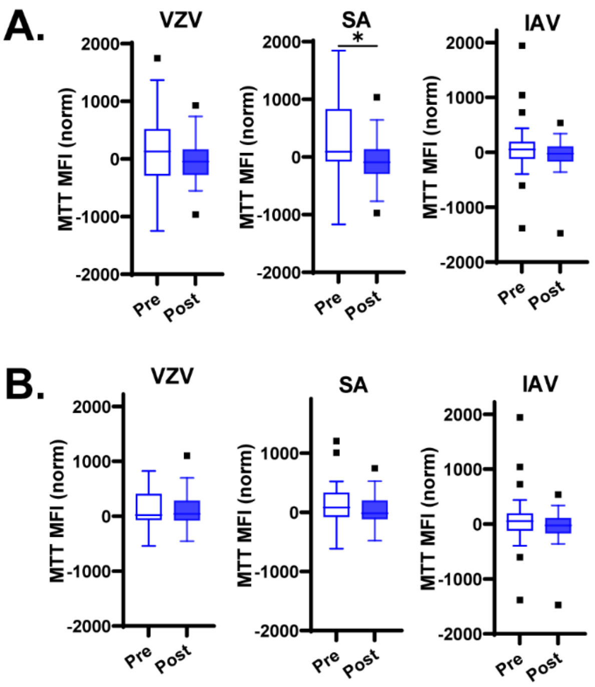

Figure S6: Active mitochondrial mass is similar in antigen-stimulated pre- and post-COVID AIM+ memory T cells.

A.) Mitochondrial mass (MTT) is similar in pre- and post-COVID AIM+ CD4+CD45RO+ memory T cells, with the exception of SA stimulated T cells. B.) MTT is similar across all antigen stimulation conditions in AIM+CD8+CD45RO+ memory T cells.

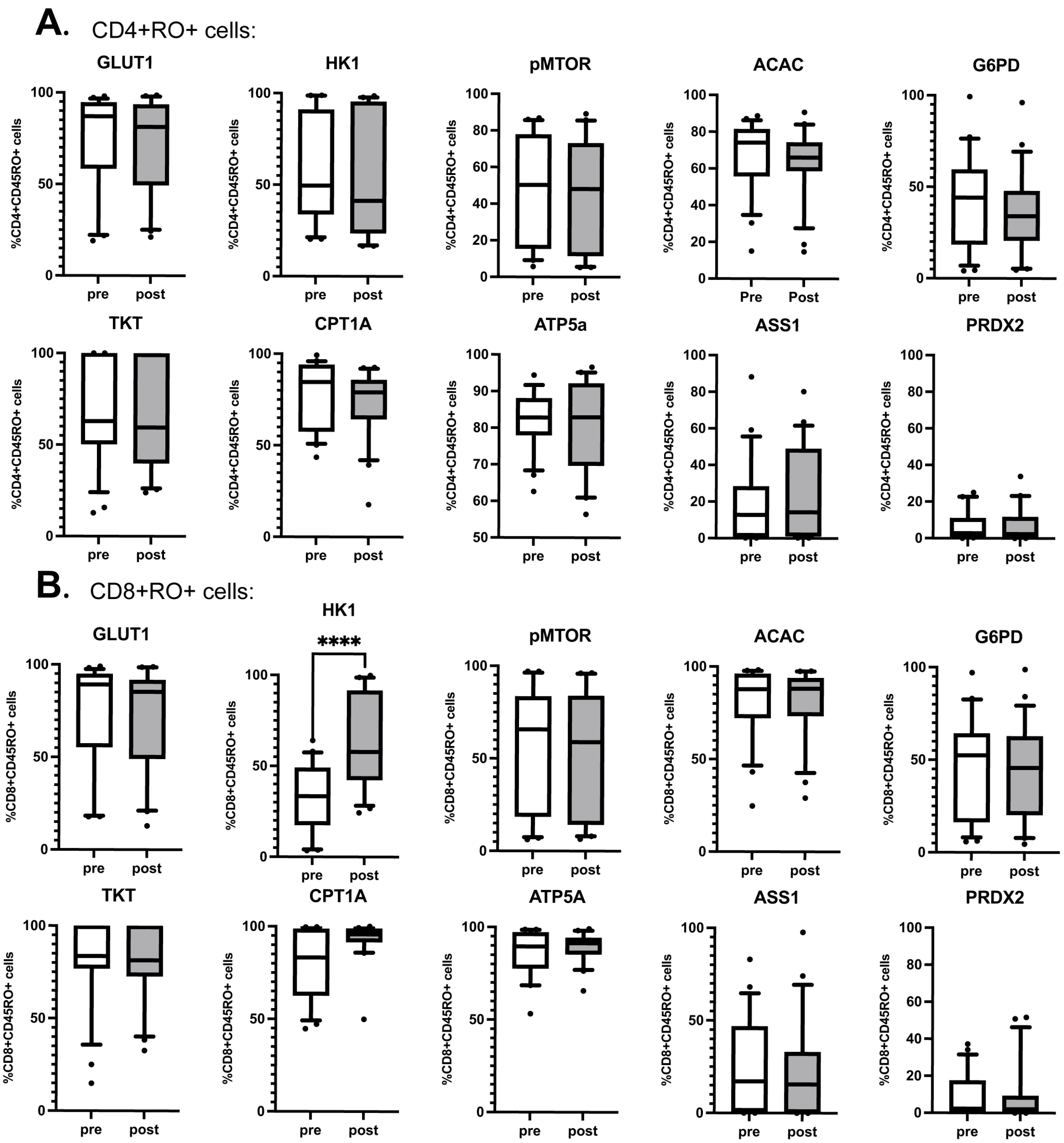

Figure S7: Met-Flow enzyme expression levels are similar in unstimulated pre- and post-COVID samples in memory T cells.

A.) %CD4+CD45RO+ T cells positive for specified marker. B.) %CD8+CD45RO+ T cells positive for specified marker. ****$p<0.001$ by paired Student’s t test.

Sample Number	N1/N2-FAM (Average Ct value)	RNAseP-HEX (Average Ct value)
1	Undetermined	25.52261743
2	Undetermined	25.20936672
3	Undetermined	26.56090885
4	Undetermined	25.90359116
5	Undetermined	26.72220572
6	Undetermined	25.69578855
7	Undetermined	24.4687618
8	Undetermined	26.33702163
9	Undetermined	24.49120691
10	Undetermined	27.93473484
11	Undetermined	27.27596799
12	Undetermined	25.04734099
13	Undetermined	25.25944452
14	Undetermined	28.32206725
15	Undetermined	25.71945025
16	Undetermined	22.72216465
17	Undetermined	27.65036109
18	Undetermined	24.70026042
19	Undetermined	26.77548932
20	Undetermined	26.52874571
21	Undetermined	23.38565904
22	Undetermined	23.80516391
23	Undetermined	27.80700817
24	Undetermined	23.66198985
25	Undetermined	23.80418968
26	Undetermined	27.12652972
27	Undetermined	22.44943011
28	Undetermined	27.7971725
29	Undetermined	24.9038592
30	Undetermined	27.0681399
31	Undetermined	27.29573103
pos. ctrl	24.07505	Undetermined
neg. ctrl	Undetermined	Undetermined

Figure S8: RT-qPCR cannot detect residual SARS-CoV-2 in post-COVID PBMCs.

RT-qPCR detected RNA encoding SARS-CoV-2 Nucleocapsid protein within RNA isolated from post-COVID PBMCs.
